# Comparisons of Anti-dsDNA Antibody Detection Methods by Chemiluminescent Immunoassay and Enzyme-Linked Immunosorbent Assay in Systemic Lupus Erythematosus

**DOI:** 10.3390/diagnostics11111940

**Published:** 2021-10-20

**Authors:** Huang-Chen Chang, Yen-Ching Wu, Jun-Peng Chen, Yi-Da Wu, Wen-Nan Huang, Yi-Hsing Chen, Yi-Ming Chen

**Affiliations:** 1 Division of Allergy, Immunology and Rheumatology, Taichung Veterans General Hospital, Taichung 40705, Taiwan; jhjean@vghtc.gov.tw (H.-C.C.); yencwu@vghtc.gov.tw (Y.-C.W.); bagidr1@hotmail.com (Y.-D.W.); gtim5555@yahoo.com (W.-N.H.); ysanne@vghtc.gov.tw (Y.-H.C.); 2 Department of Medical Research, Taichung Veterans General Hospital, Taichung 40705, Taiwan; pippan7676@vghtc.gov.tw; 3 School of Medicine, College of Medicine, National Yang Ming Chiao Tung University, Taipei 112, Taiwan; 4 College of Medicine, National Chung Hsing University, Taichung 402, Taiwan; 5 College of Business and Management, Ling Tung University, Taichung 408284, Taiwan; 6 Rong Hsing Research Center for Translational Medicine, National Chung Hsing University, Taichung 402, Taiwan; 7 Ph.D. Program in Translational Medicine, National Chung Hsing University, Taichung 402, Taiwan

**Keywords:** systemic lupus erythematosus, anti-dsDNA antibody, chemiluminescent immunoassay, enzyme-linked immunosorbent assay, lupus nephritis

## Abstract

This study aimed to compare the test results of anti-double-stranded DNA (anti-dsDNA) antibodies obtained using chemiluminescent immunoassay (CIA) and enzyme-linked immunosorbent assay (ELISA), and investigate predictors of inconsistent results. This retrospective study included 502 patients who underwent CIA and ELISA to determine their anti-dsDNA antibody values within a year. We compared the diagnostic power for SLE, disease activity, and predictive power for lupus nephritis (LN). A multivariate analysis was performed to determine the predictors of inconsistencies. CIA and ELISA were moderately correlated in terms of their consistency (Cronbach’s α = 0.571), and yielded comparably favorable results in terms of SLE diagnostic power and SLE disease activity. However, if the patient had LN, CIA displayed higher predictive power than ELISA (0.620 vs. 0.555, *p* = 0.026). Compared with the CIA/ELISA double-positive group, the inconsistent group had lower anti-C1q circulating immune complexes (CIC) antibody values (OR: 0.42, 95% CI: 0.18–0.94, *p* = 0.036), and lower SLEDAI scores (≥4) (OR: 0.33, 95% CI: 0.14–0.79, *p* = 0.013). Anti-dsDNA antibody detection with CIA exhibited higher predictability for diagnosing LN than did ELISA. In the event of inconsistencies between anti-dsDNA methods, SLE disease activity and CIC test values should be considered simultaneously.

## 1. Introduction

Systemic lupus erythematosus (SLE) is a severe autoimmune disease that produces various antibodies and involves multiple organs [1]. Among patients with SLE, 60% develop lupus nephritis (LN), which is also a crucial reason for the increased mortality of SLE [2]. Since 1982, anti-double-stranded DNA (anti-dsDNA) antibodies have been listed as diagnostic criteria for SLE by the American College of Rheumatology (ACR) [3], and studies have noted a high correlation between anti-dsDNA antibodies and LN [4]. Furthermore, anti-dsDNA antibodies are relatively effective indicators for monitoring SLE disease activity [5]. Therefore, rheumatologists have relied on anti-dsDNA antibodies to adjust medication and treatment strategies for patients with SLE.

Current methodologies for detecting anti-dsDNA antibodies include Farr radioimmunoassay, Crithidia luciliae indirect immunofluorescence test (CLIFT), enzyme-linked immunosorbent assay (ELISA), fluoroenzyme immunoassay (FEIA), and chemiluminescent immunoassay (CIA) [6]. Although the Farr radioimmunoassay has high sensitivity and specificity [7], it is rarely used clinically due to its use of radioactive materials [8,9]. CLIFT involves using the kinetoplast of *Crithidia luciliae* to form a specific combination with anti-dsDNA antibodies, making it highly specific. However, its sensitivity is lower than that of other methods, particularly in detecting early SLE [9], rendering it unsuitable as a screening test. Moreover, CLIFT is limited by qualitative inspections, requires manual interpretation, and is prone to differences due to microscope equipment, making it difficult to be used as a method for disease activity monitoring. Therefore, ELISA and CIA are preferred for clinical monitoring of disease activity [8,10,11].

The current quantitative methods used for the clinical detection of anti-dsDNA antibodies all use Wo/80 as the standard [12]; however, the poor consistency between the various methodologies, which leads to different affinity of antigens and antibodies, led to notable inconvenience in clinical use [6]. Some studies have compared the consistency of other methods with that of ELISA, but the consistency of ELISA tests was very poor [8,13]. CIA is used to detect anti-dsDNA antibodies, and despite studies noting its higher sensitivity and specificity than ELISA [14], the correlation between ELISA/CIA and the Systemic Lupus Erythematosus Disease Activity Index (SLEDAI) was only modest [14]. In addition, studies on the use of CIA in disease diagnosis, disease activity of SLE, and whether SLE invades the kidneys are rare [14,15]. Moreover, it remained unclear whether clinical parameters affect the discrepancy between ELISA and CIA. Therefore, the inconsistency of different methodologies has contributed to difficulties in clinical application [16].

Because the method of detecting anti-dsDNA antibodies in our hospital laboratory was changed from ELISA to CIA in November 2020, to study the difference between the two detection methods, we retrospectively compared the consistency between ELISA and CIA in detecting anti-dsDNA antibodies. We also determined the differences between the two methods in the clinical efficacy of SLE diagnosis, LN identification, and SLE disease activity, as well as the predictors for discrepancy in results. Identification of the relevant factors may serve as reference for clinical diagnosis and laboratory evaluation methods.

## 2. Materials and Methods

### 2.1. Study Participants

This retrospective study included 502 patients who regularly visited the Rheumatology Clinic of Taichung Veterans General Hospital and underwent examination for anti-dsDNA antibodies between November and December 2020. Of these patients, 410 were diagnosed with SLE, and met the diagnostic criteria for SLE by ACR in 1997 or the Systemic Lupus International Collaborating Clinics in 2012 [3,17,18]. The remaining 92 patients had other autoimmune diseases, which included Sjogren’s syndrome, rheumatoid arthritis, mixed connective tissue disease, systemic sclerosis, dermatomyositis, and polymyositis, all of whom met the diagnosis criteria of the ACR and European League Against Rheumatism [19,20,21,22,23,24,25]. Patients younger than 20 years old and those who did not undergo ELISA for anti-dsDNA antibody detection were excluded.

This study was approved by the Ethics Committee of Clinical Research, Taichung Veterans General Hospital (CE21255B). As patient data were anonymized before analysis, the requirement to obtain written consent from the patients was waived.

### 2.2. Study Design

The anti-dsDNA antibody values of patients who underwent CIA examination were compared with those of the same patients receiving ELISA examination between November and December 2020. The patients were divided into three groups: two with consistent results between CIA and ELISA (double-negative and double-positive) and one with inconsistent results. Patients’ age, gender, laboratory test results, disease classification, and drug use were analyzed and compared between groups.

In addition, we analyzed the correlation between CIA and ELISA and compared their sensitivity and specificity in detecting diseases to determine whether they displayed any significant differences in SLE disease diagnosis, SLE disease activity stage (SLEDAI ≥ 4), and LN (urine protein/creatinine ratio (UPCR) > 500 mg/g). Subsequently, CIA was used as the standard to conduct the multivariate analysis and identify possible predictors.

### 2.3. Measurement of Anti-dsDNA Antibody

#### 2.3.1. CIA

Anti-dsDNA antibody detection with CIA was performed by using the QUANTA Flash dsDNA (Inova Diagnostics, CA, USA), a fully quantitative test operated on the BIO-FLASH instrument (Biokit, Barcelona, Spain). The antigen used was a synthetic antigen coated onto paramagnetic beads. With the original manufacturer’s buffer and isoluminol conjugate, fitted with a luminometer, the RUL obtained was proportional to the strength of the antibody. The instrument was operated per the manufacturer’s instructions. system has recently been described [15]. Characteristics of the CIA are summarized in Table 1.

#### 2.3.2. ELISA

Anti-dsDNA antibody detection with ELISA, which used the QUANTA Lite dsDNA (Inova Diagnostics) reagent, is a semiquantitative detection of the dsDNA content in human serum, and highly purified calf thymus dsDNA was used as the antigen. The assays were performed according to the manufacturer’s instructions. The characteristics of ELISA are presented in Table 1.

### 2.4. Clinical Parameters and Lab Data

Serum tests included general biochemical parameters such as creatinine and UPCR; immune items included antinuclear antibodies (ANA), complement 3 (C3), complement 4 (C4), and anti-C1q circulating immune complexes (CIC) antibody. All operations regarding the inspection items were in accordance with the original manufacturer’s manual.

Creatinine and UPCR were determined using a spectrophotometry assay (Labospect 008, Hitachi, Tokyo, Japan); a creatinine result > 1.4 mg/dL was categorized as positive, and UPCR > 500 mg/g was considered active LN. ANA was detected using indirect immunofluorescence assay on Hep-2 cells (Inova Diagnostics); titer was detected with the NOVA View automated fluorescence microscope; a titer of ≥1:160 was considered positive and ≥1:640 was considered strongly positive. The patterns were interpreted by a senior medical examiner. C3 and C4 complement levels were determined using a turbidimetric assay (Beckman Coulter DxC 700 AU, Brea, CA, USA). C3 levels < 87 mg/dL indicated C3 hypocomplementemia, and C4 levels < 19 mg/dL indicated C4 hypocomplementemia. CIC was determined using ELISA (Inova Diagnostics), with ≥10.8 μg Eq/mL categorized as positive.

### 2.5. SLEDAI

Between September 2020 and February 2021, the disease activity of patients with SLE was evaluated according to the SLE Disease Activity Index 2000 (SLEDAI-2K) scores assessed using the CIA when detecting anti-dsDNA antibody levels [26]. After deducting the anti-dsDNA antibody score, a SLEDAI score of ≥4 was classified as having high disease activity.

### 2.6. Pharmacologic Therapy

We also analyzed whether patients had taken glucocorticoids, hydroxychloroquine, cyclophosphamide, mycophenolic acid, azathioprine, methotrexate, or cyclosporine within 6 months of the CIA test for anti-dsDNA antibodies.

### 2.7. Statistical Analysis

The demographic data of continuous parameters are shown as median (interquartile range, IQR); and for categorical variables as the number of patients. The chi-square test and Kruskal–Wallis test were used to compare age, laboratory data, disease, and drug use between groups. Cronbach’s α and receiver operating characteristics (ROC) analysis were used to analyze the consistency and discriminatory ability of two immunoassays. Logistic regression multivariate analysis was used to investigate factors associated with inconsistency between the two detection methods. All data were analyzed using SPSS version 22.0. Significance was set at *p* < 0.05.

## 3. Results

### 3.1. Patient Characteristics of Double-Positive, Double-Negative, and Inconsistent Groups

Compared with the other two groups, the double-positive group was younger; had a higher proportion of women; exhibited higher UPCR, ANA, titer ratio, and homogeneous ratio; had lower C3 and C4 levels; and had higher CIC and SLEDI scores (Table 2). The double-positive group had a higher intake rate of hydroxychloroquine, mycophenolic acid, and azathioprine than the double-negative group within 3 months (Table 2). These findings indicated that double-positive results of CIA and ELISA could effectively distinguish patients with SLE, that the patients’ disease activity was higher than the other two groups, and that they were also receiving active treatment.

### 3.2. Consistency of CIA and ELISA and Diagnostic Accuracy of SLE, High Lupus Activity, and Active LN

**Figure 1:**
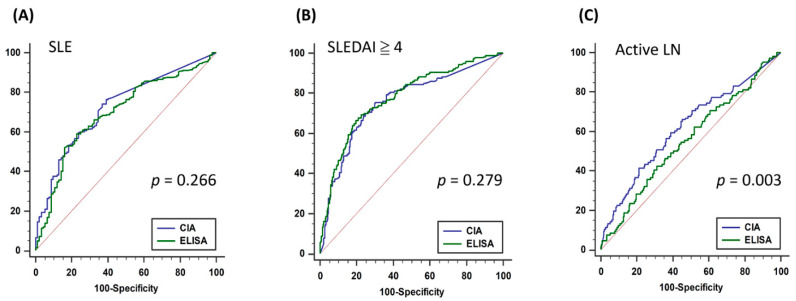
ROC analysis of (**A**) SLE, (**B**) high lupus disease activity, and (**C**) active lupus nephritis by ELISA and CIA methods. SLE: systemic lupus erythematosus; SLEDAI: Systemic Lupus Erythematosus Disease Activity Index; LN: lupus nephritis; ELISA: enzyme-linked immunosorbent assay; CIA: chemiluminescent immunoassay.

CIA and ELISA had a Cronbach’s α of 0.571 in terms of their method consistency (Table 3), indicating a moderate correlation. To compare the diagnostic power of the two methods for SLE, the disease activity of lupus erythematosus (SLEDAI ≥ 4) and the performance of LN, we performed an ROC analysis for the CIA and ELISA methods (Table 4). The results revealed that the two methodologies performed equally well for diagnosing SLE and the active stage of the disease (Figure 1A,B). Notably, when SLE was diagnosed and the patient had LN (UPCR > 500 mg/g), CIA yielded a significantly more favorable performance than ELISA (*p* = 0.003, Figure 1C). This indicated that CIA had the same effect as ELISA in diagnosing SLE and the active stage of lupus erythematosus; however, it achieved more favorable results than ELISA in the clinical prediction of LN.

### 3.3. Predictors for Inconsistent Results between CIA and ELISA

To understand the reasons for the inconsistency of the two methods, we used logistic regression to analyze the factors related to inconsistency between the dsDNA CIA and ELISA in the double-positive and inconsistent groups (Table 5). The results revealed high CIC (OR: 0.42, 95% CI: 0.18–0.94, *p* = 0.036), ELISA values (OR: 0.98, 95% CI: 0.98–0.99, *p* < 0.001), and SLEDAI ≥ 4 (OR: 0.33, 95% CI: 0.14–0.79, *p* = 0.013); in other words, the results of the two methods were relatively consistent. Our results indicated that in SLE patients with high disease activity or LN, anti-dsDNA antibodies by CIA and ELISA would exhibit more consistent results.

## 4. Discussion

Anti-dsDNA antibody detection is essential for the diagnosis and monitoring of SLE disease activity. However, different laboratory methods yield considerably different results, which often leads to clinical misinterpretation. In this study, we discovered that CIA and ELISA exhibited a moderate correlation in terms of their result consistency and that both had equally favorable results in diagnosing SLE and disease activity, indicating that they can be used to identify patients with SLE and their disease active stage. However, if the patient had LN, CIA achieved a more favorable predictive effect. Our results indicated that the consistency between ELISA and CIA was high in patients with SLE and high disease activity. Our study demonstrated that in addition to eliminating the possibility of non-SLE, SLE disease activity, presence of proteinuria, and CIC levels should be considered to facilitate the interpretation of the differences caused by the two methodologies. These results would be a fundamental guidance for clinicians to establish treatment strategies in the transition of anti-dsDNA antibody detection methods.

An Italian study [15] analyzed the correlations of commonly used methods currently available on the market, and discovered that they exhibited considerable differences, with kappa values ranging from 0.17 to 0.42. A comparison of ELISA and CIA revealed that they shared similar results in their area under the curve (0.90 and 0.79, respectively). However, studies have not examined the factors related to the inconsistent results of CIA and ELISA. Although Bentow et al. [14] discovered that in patients with high disease activity, the value of CIA was higher than that of ELISA, their study did not directly compare the two methods. No study has comprehensively compared SLE diagnosis, disease activity, and LN. By using logistic regression, we determined the predictors for the inconsistency between the two methods. Moreover, we discovered that anti-dsDNA antibody detection using CIA had a higher correlation with LN than that using ELISA. This may be because the bound anti-dsDNA antibodies in CIA were those with higher affinity [26,27], or the complexes of these dsDNA antibodies were selectively deposited in the kidneys [28]. Another possible reason was the different cleaning buffers used by the two methods, enabling CIA to retain high-binding antibodies; thus, the anti-dsDNA antibodies detected using CIA may have a greater correlation with the cause of LN [6]. Moreover, the differences in fixation method, antigen concentration, sample dilution concentration, secondary antibodies, and washing conditions may affect the results of the two methods [6]; however, this requires support from more experiments and research in the future.

We discovered that in addition to the methodological differences between ELISA and CIA, some patient-related factors may also affect the test results. We observed that if a patient had low disease activity (SLEDAI < 4) and CIC value, the two methodologies were more likely to be inconsistent; this finding has not been published before. Anti-dsDNA antibodies and CIC are essential noninvasive tests in the assessment of LN activity [29,30]. Our results demonstrated that patients with high SLEDAI and CIC had higher consistency between CIA and ELISA results. This may be due to the higher serum concentration of anti-dsDNA antibodies when SLE severity was high, therefore it would not be difficult to detect anti-dsDNA antibodies by either method used. Our result indicated that in the event of inconsistent results, clinicians should also consider the patients’ disease activity and CIC results to determine the patient’s disease status. Our findings can be an essential reference for clinical laboratories to cope with inconsistent anti-dsDNA antibody results in changing test methodology.

LN, which accounts for 60% of the patients with SLE, increases SLE mortality risk [31]. Renal biopsy is an invasive procedure required to obtain histopathology exams and accurately classify LN. Thus, UPCR and urine sediments are used for the clinical evaluation of LN. However, despite previous suggestions for using anti-dsDNA antibodies for evaluating LN due to the lack of serologically effective biomarkers [30], the considerable discrepancy in different methodologies of anti-dsDNA antibodies resulted in relatively low consistency. Nevertheless, we discovered that anti-dsDNA antibodies detected using CIA can be a predictor of LN, and ROC analysis indicated that the optimal cutoff value should be 28 IU/mL, which was within the indeterminate range (27–35 IU/mL). Our study suggested that in SLE patients with overt proteinuria (UPCR > 500 mg/g), the reference value of anti-dsDNA antibody should be revised from 35 IU/mL to 28 IU/mL. By contrast, urine tests should be performed to early detect renal involvement in SLE patients with an anti-dsDNA antibodies level > 28 IU/mL by CIA.

This study had several limitations. First, because of the retrospective design, missing data could not be avoided. Second, we did not compare the results of CLIFT and FEIA; thus, our conclusion cannot be extrapolated to other detection methods. Meanwhile, the optimized cut-offs in our study were determined by a statistical analysis of the test results from a study cohort of SLE and non-SLE patients, so the values of borderline/indeterminate and strong positive cut-offs could not be measured. Third, the time points for CIA and ELISA detection were not the same. We could not preclude the variations of anti-dsDNA antibodies and the effect of drug treatment during this time period between two tests. Finally, the impact of clinical manifestations other than LN to anti-dsDNA antibody test results remained unknown, and deserve further investigation.

## 5. Conclusions

Our real-world, hospital-based study discovered that the anti-dsDNA antibodies detected by CIA and ELISA were similar in the diagnosis of SLE and active lupus disease activity. However, CIA exhibited a higher predictability in LN than in ELISA. In patients with high SLE disease activity and CIC levels, the two methods displayed favorable consistency. Our findings provide a valuable guidance for clinicians to interpretate laboratory results and establish therapeutic strategies for patients with SLE in the event of switching test methodology.

## Figures and Tables

**Table 1:** Comparisons of anti-dsDNA antibody detection methods by ELISA and CIA.

Characteristic	QUANTA Lite^®^ dsDNA	QUANTA Flash dsDNA
Technology	ELISA	CIA
Manufacturer	Inova Diagnostics	Inova Diagnostics
Detection	Semi-Quantitative	Quantitative
Assay time (minutes)	90	30
Analytical measuring range	0–>370.5 WHO units/ml	9.8–666.9 IU/mL
Cut-off value (range)	Negative 0–92.6 Equivocal 92.7–138.9 Moderate Positive 139–370.4 Strong Positive > 370.5	Negative 9.8–27 Indeterminate 27–35 Positive > 35
Antigen source	Calf thymus dsDNA	Synthetic dsDNA

Anti-dsDNA: anti-double-stranded DNA; ELISA: enzyme-linked immunosorbent assay; CIA: chemiluminescent immunoassay.

**Table 2:** Comparisons of demographic data and patient characteristics among three groups of anti-dsDNA antibody positivity detected using ELISA and CIA.

	Double-Negative (*n* = 259)		Inconsistent (*n* = 102)		Double-Positive (*n* = 141)		*p*-Value
Age	47.6	(37.8–58.9)	45.6	(37.1–54.6)	42.0	(33.6–49.7)	<0.001 *
Gender							0.013 *
Female	214	(82.6%)	90	(88.2%)	131	(92.9%)	
Male	45	(17.4%)	12	(11.8%)	10	(7.1%)	
Disease							<0.001 *^#$^
SLE	185	(71.4%)	93	(91.2%)	132	(93.6%)	
Non-SLE	74	(28.6%)	9	(8.8%)	9	(6.4%)	
Lab data							
Creatinine (mg/dL)							0.375
<1.4	220	(85.3%)	84	(82.4%)	125	(88.7%)	
≥1.4	38	(14.7%)	18	(17.6%)	16	(11.3%)	
UPCR (mg/g)							0.019 *
<500	167	(78.8%)	71	(74.0%)	90	(65.2%)	
≥500	45	(21.2%)	25	(26.0%)	48	(34.8%)	
ANA							<0.001 *
<1:80	6	(3.6%)	3	(4.3%)	4	(4.3%)	
1:80–1:640	98	(59.4%)	32	(46.4%)	29	(31.2%)	
≥1:640	61	(37.0%)	34	(49.3%)	60	(64.5%)	
Homogeneous (*n* = 281)	78	(43.6%)	28	(58.3%)	38	(70.4%)	0.001 *
C3 (mg/dL)							<0.001 *^#^
<87	34	(13.5%)	38	(37.6%)	85	(60.3%)	
≥87	217	(86.5%)	63	(62.4%)	56	(39.7%)	
C4 (mg/dL)							<0.001 *^#^
<19	63	(25.2%)	48	(47.5%)	94	(66.7%)	
≥19	187	(74.8%)	53	(52.5%)	47	(33.3%)	
CIC (μg Eq/mL)							<0.001 *^#^
<10.8	140	(90.3%)	62	(72.1%)	66	(53.2%)	
≥10.8	15	(9.7%)	24	(27.9%)	58	(46.8%)	
CIA (IU/mL)	9.8	(9.8–15.9)	45.6	(22.5–85.7)	140.5	(78.2–254.8)	<0.001 *^#$^
ELISA (WHO units/mL)	18.1	(10.1–42.4)	106.7	(66.1–177.3)	284.6	(207.3–379.2)	<0.001 *^#$^
SLEDAI	0.0	(0.0–2.0)	2.0	(0.0–4.0)	4.0	(4.0–8.0)	<0.001 *^#$^
Drug							
Glucocorticoid	215	(83.0%)	93	(91.2%)	132	(93.6%)	0.004 *
Hydroxychloroquine	201	(77.6%)	93	(91.2%)	133	(94.3%)	<0.001 *
Cyclophosphamide	57	(22.0%)	35	(34.3%)	42	(29.8%)	0.036
Mycophenolic acid	57	(22.0%)	57	(40.4%)	36	(35.3%)	<0.001 *
Azathioprine	117	(45.2%)	65	(63.7%)	106	(75.2%)	<0.001 *
Methotrexate	63	(24.3%)	21	(20.6%)	34	(24.1%)	0.738
Cyclosporin	47	(18.1%)	25	(24.5%)	36	(25.5%)	0.163

Kruskal–Wallis test. Post hoc analysis, * double-negative vs. double-positive; ^#^ double-negative vs. inconsistent; ^$^ double-positive vs. inconsistent, *p* < 0.05 Anti-dsDNA: anti-double-stranded DNA; UPCR: urine protein/creatinine ratio; ANA: anti-nuclear antibodies; CIC: anti-C1q circulating immune complexes antibody; CIA: chemiluminescent immunoassay; ELISA: enzyme-linked immunosorbent assay; SLEDAI: Systemic Lupus Erythematosus Disease Activity Index.

**Table 3:** Consistency analysis of anti-dsDNA antibody tests by ELISA and CIA.

	CIA				Kappa Value
	Negative		Positive		
ELISA					0.571
Negative	259	(51.6%)	65	(12.9%)	
Positive	37	(7.4%)	141	(28.1%)	

Anti-dsDNA: anti-double-stranded DNA; CIA: chemiluminescent immunoassay; ELISA: enzyme-linked immunosorbent assay.

**Table 4:** Comparisons of diagnostic accuracies of SLE, high lupus activity, and active lupus nephritis of two anti-dsDNA antibody detection methods.

Outcome: SLE, *n* = 502									
**Variables**	**AUC**	(95% CI)	*p*-Value	Optimal Cutoff	Sensitivity	Specificity	Accuracy	PPV	NPV
CIA	0.723	(0.682–0.762)	<0.001	>10.1	76.3%	60.9%	73.5%	89.7%	36.6%
ELISA	0.696	(0.654–0.736)	<0.001	>61.4	59.0%	77.2%	62.4%	92.0%	29.7%
Outcome: SLE & SLEDAI ≥ 4, *n* = 410									
CIA	0.757	(0.712–0.797)	<0.001	>40.3	70.0%	75.2%	72.9%	68.9%	76.2%
ELISA	0.777	(0.734–0.817)	<0.001	>133.7	66.7%	80.4%	74.4%	72.7%	75.5%
Outcome: SLE & UPCR ≥ 500, *n* = 410									
CIA	0.620	(0.570–0.668)	<0.001	>28.0	66.0%	54.8%	57.8%	34.7%	81.6%
ELISA	0.555	(0.505–0.605)	0.095	>166.2	42. 5%	68.8%	61.8%	33.1%	76.7%

Anti-dsDNA antibodies: anti-double-stranded DNA antibodies; CIA: chemiluminescent immunoassay; ELISA: enzyme-linked immunosorbent assay; SLE: systemic lupus erythematosus; UPCR: urine protein/creatinine ratio; AUC: area under curve; PPV: positive predictive value; NPV: negative predictive value.

**Table 5:** Logistic regression of risk factors for inconsistency of anti-dsDNA antibody tests by ELISA and CIA.

	Univariate			Multivariable			
	Odds Ratio	95%CI	*p*-Value	Odds Ratio	95%CI		*p*-Value
Age	1.03	(1.01–1.05)	0.010 *				
Female	Reference						
Male	1.75	(0.72–4.22)	0.215				
Creatine < 1.4 (mg/dL)	Reference						
Creatine ≥ 1.4 (mg/dL)	1.67	(0.81–3.47)	0.165				
UPCR < 500 (mg/g)	Reference						
UPCR ≥ 500 (mg/g)	0.66	(0.37–1.17)	0.157				
ANA < 1:80	Reference						
ANA 1:80–1:640	1.47	(0.30–7.14)	0.632				
ANA ≥ 1:640	0.76	(0.16–3.58)	0.724				
ANA Homogeneous (*n* = 281)	0.59	(0.26–1.34)	0.206				
C3 ≥ 87 (mg/dL)	Reference			Reference			
C3 < 87 (mg/dL)	0.40	(0.24–0.67)	0.001 **	0.93	(0.40–2.13)		0.861
C4 ≥ 19 (mg/dL)	Reference			Reference			
C4 < 19 (mg/dL)	0.45	(0.27–0.77)	0.003 **	1.04	(0.43–2.51)		0.930
CIC < 10.8 (μg Eq/mL)	Reference			Reference			
CIC ≥ 10.8 (μg Eq/mL)	0.44	(0.24–0.79)	0.006 **	0.42	(0.18–0.94)		0.036 *
Anti-dsDNA antibody by CIA (IU/mL)	0.98	(0.98–0.99)	<0.001 **				
Anti-dsDNA antibody by ELISA (WHO units/mL)	0.98	(0.98–0.99)	<0.001 **	0.98	(0.98–0.99)		<0.001 **
SLEDAI < 4	Reference			Reference			
SLEDAI ≥ 4	0.22	(0.12–0.39)	<0.001 **	0.33	(0.14–0.79)		0.013 *

Logistic regression. * *p* < 0.05, ** *p* < 0.01. Anti-dsDNA antibodies: anti-double-stranded DNA antibodies; UPCR: urine protein/creatinine ratio; ANA: anti-nuclear antibodies; CIC: anti-C1q circulating immune complexes antibody; CIA: chemiluminescent immunoassay; ELISA: enzyme-linked immunosorbent assay; SLEDAI: Systemic Lupus Erythematosus Disease Activity Index.

## Data Availability

The aggregated data are available from the corresponding author upon reasonable request.
